# Minced Umbilical Cord Fragments as a Source of Cells for Orthopaedic Tissue Engineering: An In Vitro Study

**DOI:** 10.1155/2012/326813

**Published:** 2012-03-07

**Authors:** A. Marmotti, S. Mattia, M. Bruzzone, S. Buttiglieri, A. Risso, D. E. Bonasia, D. Blonna, F. Castoldi, R. Rossi, C. Zanini, E. Ercole, E. Defabiani, C. Tarella, G. M. Peretti

**Affiliations:** ^1^Department of Orthopaedics and Traumatology, University of Turin, Mauriziano Hospital, Largo Turati 62, 10128 Turin, Italy; ^2^Molecular Biotechnology Center, University of Turin, Via Nizza 52, 10126 Turin, Italy; ^3^Research & Development Department, EuroClone S.p.A., Molecular Biotechnology Center, University of Turin, Via Nizza 52, 10126 Turin, Italy; ^4^Department of Obstetrics and Gynaecology, University of Turin, Mauriziano Hospital, Largo Turati 62, 10128 Turin, Italy; ^5^Department of Haematology and Cell Therapy Division, University of Turin, Mauriziano Hospital, Largo Turati 62, 10128 Turin, Italy; ^6^Department of Sport, Nutrition and Health Sciences, University of Milan, Via Kramer 4/A, 20129 Milan, Italy

## Abstract

A promising approach for musculoskeletal repair and regeneration is mesenchymal-stem-cell- (MSC-)based tissue engineering. The aim of the study was to apply a simple protocol based on mincing the umbilical cord (UC), without removing any blood vessels or using any enzymatic digestion, to rapidly obtain an adequate number of multipotent UC-MSCs. We obtained, at passage 1 (P1), a mean value of 4, 2 × 10^6^ cells (SD 0,4) from each UC. At immunophenotypic characterization, cells were positive for CD73, CD90, CD105, CD44, CD29, and HLA-I and negative for CD34 and HLA-class II, with a subpopulation negative for both HLA-I and HLA-II. Newborn origin and multilineage potential toward bone, fat, cartilage, and muscle was demonstrated. Telomere length was similar to that of bone-marrow (BM) MSCs from young donors. The results suggest that simply collecting UC-MSCs at P1 from minced umbilical cord fragments allows to achieve a valuable population of cells suitable for orthopaedic tissue engineering.

## 1. Introduction

The repair and regeneration of bone, articular cartilage, and muscle are a major challenge in biomedical research. One of the most promising approaches is represented by mesenchymal stem-cell-based tissue engineering. Mesenchymal stem cells (MSCs) have been under constant investigation since the 1990s [1] for their excellent proliferation potential and their capability for differentiation into multiple lineages. Moreover, their immunosuppressive properties make them a suitable candidate for allogenic cell therapy. Allogenic cell-based approaches imply MSCs to be isolated from a donor, expanded, and cryopreserved in allogenic MSC banks, providing a readily available source for cell replacement therapy.

Bone marrow (BM) represents the most commonly used source of adult MSCs. BM-MSCs have been functionally defined as plastic-adherent, nonhaematopoietic, multipotential cells that support haematopoietic stem cells expansion in vitro and that are able to differentiate into cells of various connective tissues. Various cell-surface markers have been associated with a mesenchymal phenotype, as CD105, CD73, CD90, and HLA-ABC proteins, while lack expression of CD45, CD34, CD14, or CD11b, CD79 alpha or CD19 and HLA-DR were also considered characteristic of this cell population [2]. Previous studies have extensively shown their ability to differentiate into bone [3, 4], muscle [5], adipose tissue [6], cartilage [7], and tendon [8]. Nevertheless, several limitations as the painful procedure for BM collection, the limited number of BM-MSCs available for autogenous use, and the concomitant reduction in allogeneic BM donations have raised an increasing interest in identifying alternative sources of MSCs.

Human umbilical cord (UC) has been recently suggested as a valid alternative tissue for MSCs [9]. The UC is a tissue of extraembryonic origin lying between the mother and the fetus, consisting of two arteries, one vein, intervessels connective tissue (the Wharton's jelly), and umbilical epithelium. The UC is normally discarded after birth. Therefore, UC collection does not require any invasive procedure nor implies major ethical concerns. MSCs have been isolated from all compartments of the umbilical cord tissue, namely, the umbilical vein endothelium and subendothelium and the Wharton's jelly. Within Wharton's jelly, MSCs have been isolated from three regions: the perivascular zone (UC perivascular cells), the intervascular zone, and the subamnion. MSCs can be also isolated from umbilical cord blood, but the limited amount of blood that can be collected and the technical difficulties of this procedure make umbilical cord blood less suitable than UC connective and perivascular tissues. Both Wharton's jelly-derived cells and umbilical vein perivascular cells (endothelium- and subendothelium-derived MSCs) have shown multilineage capability along with immunoregulatory properties [10, 11]. It has been shown that a single injection of MHC-mismatched unactivated human UC-MSCs did not induce a detectable immune response [12]; therefore, they can be tolerated in allogeneic transplantation [13]. These cells share with BM-MSCs several surface markers as CD73, CD90, and CD105 and did not express CD34 [14]. Moreover, UC-MSCs show low expression of HLA class I and no expression of HLA class II unless stimulated with IFN-*γ* [15, 16].

The aim of this study was to apply a simple protocol based on mincing the whole UC, without removing any blood vessels or using any enzymatic digestion, in order to obtain an adequate number of multipotent UC-MSCs at P1. This method did not imply selecting a single cell population from the different UC regions (Wharton's jelly, endothelium, and subendothelium) but allowed for accessing to a mixed population of MSCs from all UC areas. Multilineage potential of these cells, immunophenotype, origin, and telomere length were verified at P1. This study intends to identify a cell population suitable for tissue engineering applications in orthopaedics and musculoskeletal medicine with a simple method with minor cell manipulation, in order to establish a good manufacturing practice protocol for the isolation and expansion of multipotent UC-MSCs.

## 2. Materials and Methods

Approvals were obtained both from the Ethical Committee of MBC (Molecular Biotechnology Center), University of Torino, and from the Ethical Committee of Mauriziano Hospital, Torino (Italy).

### 2.1. UC Collection and Processing

After obtaining patient's own informed consent, 4 fresh UC samples of women with healthy pregnancies were retrieved at the end of gestation during caesarean deliveries from the Department of Obstetrics and Gynecology of Mauriziano Hospital (Torino, Italy).

**Figure 1:**
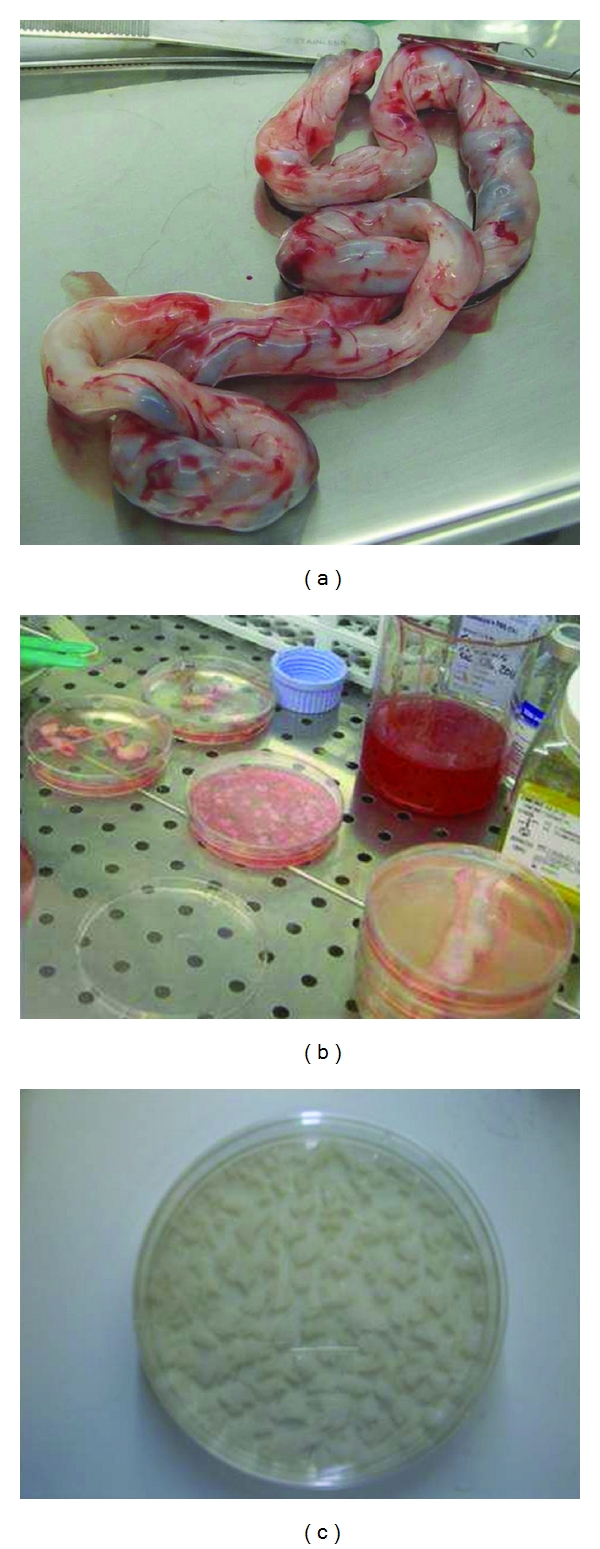
Umbilical cord processing method (a, b, c).

The UC samples (mean length 29,5 ± 4, 8 cm, range 25–35 cm, weight 30,5 ± 5, 3 g, range 25–36 g) were collected in a phosphate-buffered saline (PBS) transfer medium containing 200 mg/100 mL ciprofloxacin (Bayer, Milan, Italy), 500 IU heparin (Pharmatex, Milan, Italy), and were immediately processed. After transferring under a sterile laminar flow cell culture hood, the cord length and weight were estimated and the UC was washed twice in PBS to remove the traces of contaminant red blood cells. The UC was firstly cut into 3 cm long segments, which were subsequently cut longitudinally and split open to expose the inner surface. The UC segments were transferred to a 60 cm^2^ Petri dish (Corning, New York, NY, USA) containing 10 mL MSC expansion medium, consisting of Dulbecco's Modified Eagle Medium/F-12 (D-MEM) (Invitrogen, Carlsbad, CA, USA) enriched with 5% human platelet lysate obtained from healthy donors, 10% Fetal Bovine Serum (FBS), 1X penicillin/streptomycin, 1X sodium pyruvate, 1X nonessential amino acids (Invitrogen, Carlsbad, CA, USA), 500 IU heparin (Pharmatex, Milan, Italy). The UC segments were then manually minced into very small cuboidal fragments (4–7 mm length) using no. 15 sterile scalpels. The small UC fragments were then transferred and evenly distributed into 6-7 different 60 cm^2^ Petri dishes (approximately 40–45 fragments/Petri dish) and incubated in the MSC expansion medium at 37°C in a humidified atmosphere with 5% CO_2_ (day 0) (Figures 1(a), 1(b), and 1(c)). Fragments of UC were left undisturbed in culture and monitored for up to 2 weeks to allow identification of MSCs in the dishes.

### 2.2. Culture of UC-MSCs

After 2 weeks from the initial seeding (day 14), the UC tissue was removed and adherent cells were allowed to expand for 2 additional weeks. Forty percent of the medium was changed every 3-4 days. After this time period (day 28), the adherent cells (P0) were trypsinized, centrifuged at 1200 rpm for 10 min, resuspended in MSC expansion medium, and replated for one consecutive expansion step at a density of 100–200 cells/cm^2^, until full confluence was reached (P1). Cell confluence at P1 was reached after approximately 14 days (day 42).

At the end of P1 passage (day 42), the living cells were counted by trypan blue dye exclusion (Sigma-Aldrich, St. Louis, MO, USA).

UC-MSCs from three UC were used for immunophenotypic characterization, multilineage differentiation, and fluorescence in situ hybridization. UC-MSCs from one UC were used for telomere length analysis.

### 2.3. Immunophenotypic Characterization of UC-MSCs

Immunophenotyping of the expanded UC-MSCs was done using flow cytometry at the P1 passage of culture. 1,5 × 10^6^ UC-MSCs were used for flow cytometry.

The following antibodies were used: CD90-Peridinin Chlorophyll Protein (PerCP)-cyanine dye Cy5.5, CD105-fluorescein isothiocyanate (FITC) (Biolegend, San Diego, CA), CD73-Allophycocyanin (APC), CD34-phycoerythrin (PE), HLA-DR-FITC, HLA-PerCP, HLA-ABC-PE, CD29-APC (BD Biosciences, San Jose, CA), CD44-Alexa Fluor (Cell Signaling Technology, Danvers, MA), PE-conjugated anti-mouse immunoglobulin G (IgG) (Southern Biotechnology Associates, Birmingham, Alabama, USA), isotypematched IgG-FITC, IgG-PE and IgG-PE-Cy5 control antibodies (Biolegend, San Diego, CA).

Analysis was performed on a FACScan (Becton Dickinson (BD), Buccinasco, Italy) for at least 10.000 events and using CellQuest software (BD).

### 2.4. UC-MSCs Differentiation

UC-MSCs at P1 from three UC samples were assessed for multilineage differentiation.

The adipogenic, osteogenic, chondrogenic, and myogenic differentiation ability of UC-MSCs was determined as briefly described, following previously published methods [1, 14].

For adipogenic differentiation, 5 × 10^5^ UC-MSCs were cultured with EUROMED Adipogenic Differentiation Kit (EuroClone, Pavia, Italy) for 3 weeks. To evaluate the differentiation, cells were fixed with 4% paraformaldehyde in PBS for 20 minutes at room temperature and stained with 0.5% Oil Red O (Sigma-Aldrich, Milan, Italy) in methanol (Sigma-Aldrich, Milan, Italy) for 20 minutes at room temperature.

Osteogenic differentiation was assessed by culturing 5 × 10^5^ UC-MSCs in EUROMED Osteogenic Differentiation Kit (EuroClone, Pavia, Italy). Medium was changed twice a week for 3 weeks. To evaluate the differentiation, cells were fixed with 4% paraformaldehyde for 20 minutes and stained with Alizarin Red, pH 4.1 (Lonza, Bergamo, Italy) for 20 minutes at room temperature. Cells were also studied with Alkaline Phosphatase stain (Alkaline Phosphatase Kit-based on naphthol AS-BI and fast red violet LB, Sigma-Aldrich). For immunofluorescence analysis, we also cultured cells directly on coverslips in the same conditions to identify the presence of osteocalcin (Abcam, Cambridge, UK) [17].

A pellet culture system was used for chondrogenesis (Figures 5(a) and 5(b)). Approximately 1 × 10^6^ UC-MSCs were centrifuged in 15-mL conical polypropylene tube (Falcon BD Bioscience, Milan, Italy) at 150 g for 5 minutes and washed twice with DMEM. The pellets were cultured in EUROMED Chondrogenic Differentiation Kit (EuroClone, Pavia, Italy) supplemented with 10 ng/mL of Transforming Growth Factor *β*3 (SeroLab, Lausanne, Switzerland). Medium was changed every 3 days for 28 days. Pellets were fixed in ethanol 80% overnight, and the paraffin-embedded sections were stained for glycosaminoglycans using Safranin O and for sulfated proteoglycans with Alcian blue (Sigma-Aldrich, Milan, Italy) an with Toluidine Blue [18].

Myogenic differentiation was performed using 5 × 10^5^ UC-MSCs, plated in six-well culture plates (BD Falcon, Milan, Italy) on coverslips in DMEM with 10% knockout serum, 1% penicillin, 1% streptomycin, 0.1 mM *β*-mercaptoethanol, and 40 ng/mL fibroblast growth factor (FGF) [14]. The medium was changed every 3 days. After 21 days in culture, cells were immunostained with a monoclonal antibody against human myogenin (Abcam, Cambridge, UK).

The same osteogenic, chondrogenic, and adipogenic differentiation protocols were used on a population of BM-MSCs as a control.

### 2.5. Immunofluorescence Analysis

Immunofluorescence analysis of cells is briefly described as it follows.

The UC-MSCs grown on glass coverslips were rinsed briefly in phosphate-buffered saline (PBS 1X) and fixated with 4% paraformaldehyde in PBS pH 7.4 for 15 min at room temperature. Samples were washed three times with PBS 1X. To obtain permeabilization, samples were incubated for 1 min with PBS 1X containing 0.5% Triton X-100 and washed three times with PBS 1X. For blocking, cells were incubated with 1% BSA in PBS 1X for 1 hour to block unspecific binding of the antibodies; then samples were incubated with the primary diluted antibody (antiosteocalcin or antimyogenin, depending on the cell line) in 0.1% BSA in PBS 1X in a humidified chamber for 1 hr at room temperature (dilution 1 : 50). Then, the cells were washed three times in PBS 1X and subsequently incubated with the secondary antibody (anti-mouse conjugated with Alexa Fluor 488, Invitrogen, Milan, Italy) in 0.1% BSA for 1 hr at room temperature in dark (dilution 1 : 1000). The solution was washed three times with PBS, and, for counterstaining, cells were incubated with DAPI (4′,6-diamidino-2-phenylindole, DNA stain) for 7 min. Then, cells were rinsed with PBS 1X three times for 15 min; finally, the coverslip was mounted with a drop (5 *μ*L) of mounting medium for observation.

### 2.6. Fluorescence In Situ Hybridization

The origin of UC-MSCs was performed using a fluorescence in situ hybridization (FISH) for 2 UC samples. This was carried out in the two cases of male newborns among the four UC specimens collected. 1 × 10^5^ cells were used.

The probes used were X centromere Xp11.1-q11.1 (DXZ1) (green) and Y heterochromatin Yq12 (DYZ1) (red). The enumeration probe set contained chromosome specific DNA repeat sequences located at the centromere of chromosome X and in the heterochromatic block of chromosome Y (Cytocell Aquarius, Cambridge, UK).

Cells derived from P1 were fixed in Carnoy's fixative, according to the institutional protocol guidelines. FISH protocol was performed according to the manufacturer's instructions (Cytocell Aquarius, Cambridge, UK). Results were analyzed using fluorescence microscope (Olympus-BX41, magnification 100x, triple filter RED-GREEN-DAPI).

### 2.7. Telomere Length Analysis

Telomere length was evaluated on UC-MSCs at P1 from one UC, and results were compared to telomere length of bone marrow MSCs taken from bone marrow aspirate of 6 healthy adult volunteers (age 20–30).

Approximately, 4 × 10^6^ cells were used for telomere length analysis. This analysis was performed on one UC only. Telomere length was determined using a Southern Blot analysis as previously described [19]. 2 *μ*g of DNA were digested by mixing Hinf I (20 U) and Rsa I (20 U; Roche Diagnostic, Mannheim, Germany) and incubated at 37°C for 2 hours. Digested DNA fragments were separated by 0.8% agarose gel electrophoresis in 1X Tris-acetate-EDTA running buffer. Positively charged nylon membrane was used to transfer DNA separated by electrophoresis. After overnight transfer, the nylon membrane was exposed to ultraviolet light to fix DNA fragments. TeloTAGGG Telomere Length Assay Kit (Roche Diagnostic) was used for the hybridization phase. The membrane was submerged in a prehybridization solution for 2 hours at 62°C under gentle stirring and then incubated in the hybridization solution (2 *μ*L of the digoxigenin (DIG-) labeled telomere-specific probe added to the prehybridization solution) for 3 hours at the same temperature. After hybridization, the membrane was washed twice at room temperature in stringent wash buffer I (2X SSC, 0,1% SDS) for 10 minutes and then twice at 37°C in stringent wash buffer II (0.2X SSC, 0,1% SDS) for 20 minutes; it was incubated with blocking solution 1X for 30 minutes at room temperature and then with a DIG-specific antibody covalently coupled to alkaline phosphatase (AP). Finally, CDP Star, a chemiluminescent substrate, was dropped onto the membrane to stimulate AP to produce light emission. This emission was detected by X-ray film (Lumi-Film Chemiluminescent Detection Film, Roche Diagnostic) and scanned for analysis. Analysis was performed using Quantity One (BioRad). For each telomere smear, peak telomere restriction fragments length and the point of maximum signal intensity defining the highest concentration of telomere repeats were calculated as the median value of telomere length of the cell population examined.

### 2.8. Statistical Methods

The Student's *t*-test was used to compare telomere lengths evaluated in UC-MSCS and in healthy people bone marrow. Differences were considered significant for *P* < 0,05. Statistical analysis was carried out with the statistical software package GraphPad Prism 5.0 (GraphPad Software, San Diego, CA, USA).

## 3. Results

### 3.1. Morphologic and Immunophenotypic Characterization of UC-MSCs

**Figure 2:**
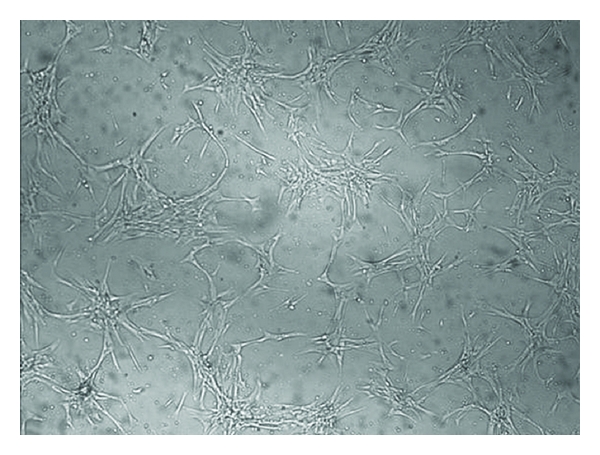
MSCs from the umbilical cord tissue during cell culture. Cell cultures (P0) 10 days after UC fragments removal (24 days of culture), magnification 10x. Cells show a fibroblast-like morphology.

In primary cultures, typical spindle-shaped adherent cells were observed migrating from the UC tissue fragments and initiating the colony formation approximately at day 14 after UC fragments seeding. After removing the UC fragments at day 14, cells at P0 took approximately 10-days period to gain 60% confluence (Figure 2), while full confluence was observed after 14 days. The UC-MSC clones (P0) were then collected at day 28 and replated for further expansion (P1). Confluence at P1 was observed after 14 days of culture (day 42).

At day 42, we obtained at P1 a mean value of 4,2 × 10^6^ cells ± 0,4 from each UC. From the initial UC fragments seeding (day 0), we obtained at the end of P1 (day 42) 0,14 × 10^6^ cells/g of UC seeded.

**Figure 3:**
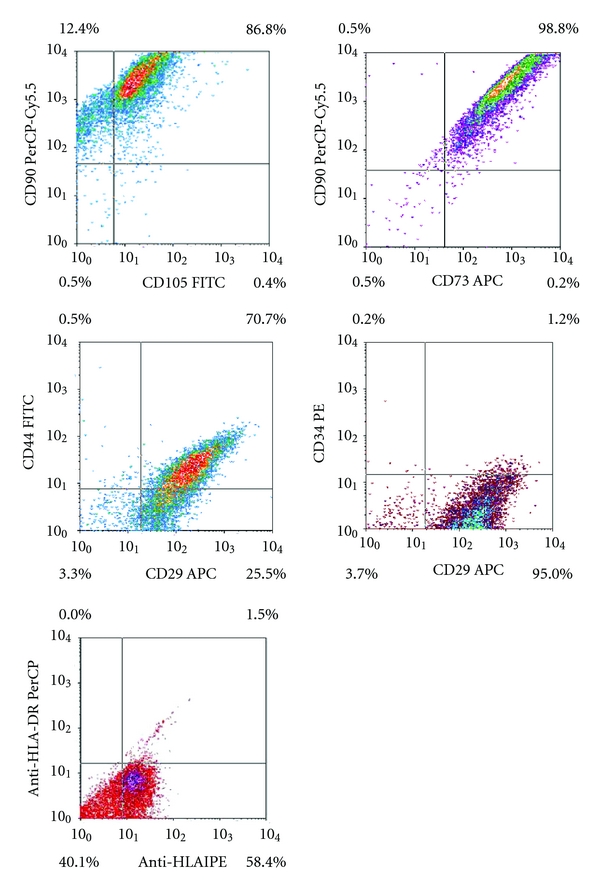
Flow cytometry analysis of surface-marker expression on umbilical cord mesenchymal cells, after one stage in culture. Flow cytometry reveals positivity for CD73, CD90, CD105, CD44, CD29 and a notable presence of negative double cells for both HLA-ABC and HLA-DR proteins.

The phenotype of UC cells was analyzed by flow cytometry. Data from one representative experiment are reported in Figure 3. The majority of collected UC cells showed a positive expression of the main MSC markers CD73, CD90, and CD105, as well as of CD44 and CD29. Furthermore, they were negative for the typical haematopoietic marker CD34.

The data also demonstrated the presence of HLA-ABC proteins and the absence of HLA-DR. Additionally, we have visualized a notable presence (40%) of negative double cells for both HLA-ABC and HLA-DR proteins.

### 3.2. Differentiation of UC-MSCs

**Figure 4:**
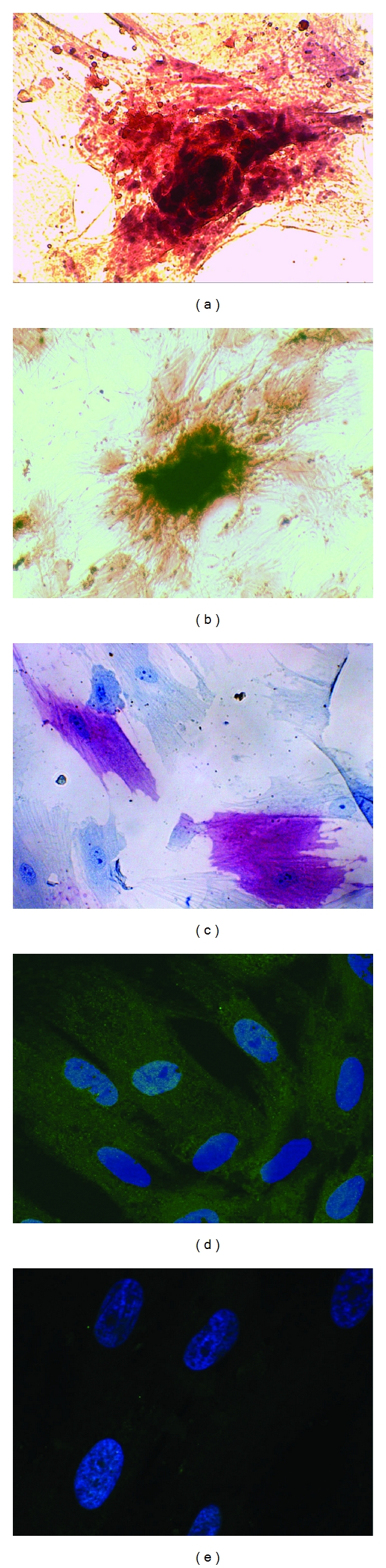
Osteogenic differentiation. Alizarin red staining revealing formation of calcium deposition in UC-MSCs (a) and in human BM-MSCs (b); (c) alcaline phosphatase cytoenzymatic staining of UC-MSCs; immunofluorescence for osteocalcin in UC-MSCs cultured in osteogenic medium; (d) osteocalcin stained with primary antibody against and with a secondary antibody anti-mouse (conjugated with Alexa Fluor 488) and merged with DAPI (blue, nuclei), (e) negative control with DAPI and secondary antibody only.

In the osteogenic-stimulated cultures, significant calcium deposition was observed with Alizarin Red staining inside the cluster of cells after 21 days, consistent with osteogenic commitment of UC-MSCs. UC-MSCs showed a pattern similar to bone-marrow MSCs after 21 days of cultures with the same medium, as it is shown in Figures 4(a) and 4(b).

Cells were also positive for alkaline phosphatase stain (Figure 4(c)).

Osteocalcin was found in cytoplasm at immunofluorescence in UC-MSCs after commitment toward osteogenic pathway (Figures 4(d) and 4(e)).

**Figure 5:**
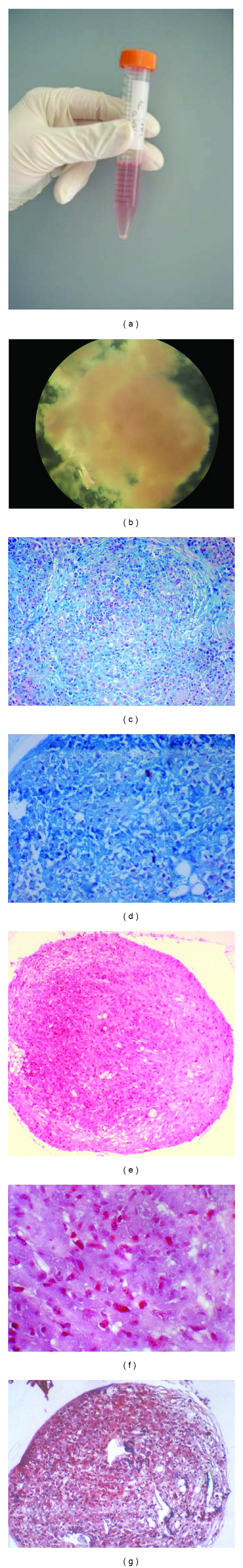
Chondrogenic differentiation. Cells growing in pellet culture system in chondrogenic medium (a, b). Histological section after chondrogenic commitment; UC-MSCs (c) and BM-MSCs (d) stained with Alcian Blue; UC-MSCs (e, f) and BM-MSCs (g) stained with Safranin O (f): higher magnification).

Chondrogenic commitment with the pellet culture system was observed at 28 days. At the histological evaluation, pellets of UC-MSCs from all three UCs exhibited positive staining for Alcian Blue and Safranin O (Figure 5) and Toluidine blue methods (data not shown). UC-MSCs showed roundish shape and a pattern similar to bone-marrow MSCs cultured in the same conditions, as it is shown in the Figure 5.

**Figure 6:**
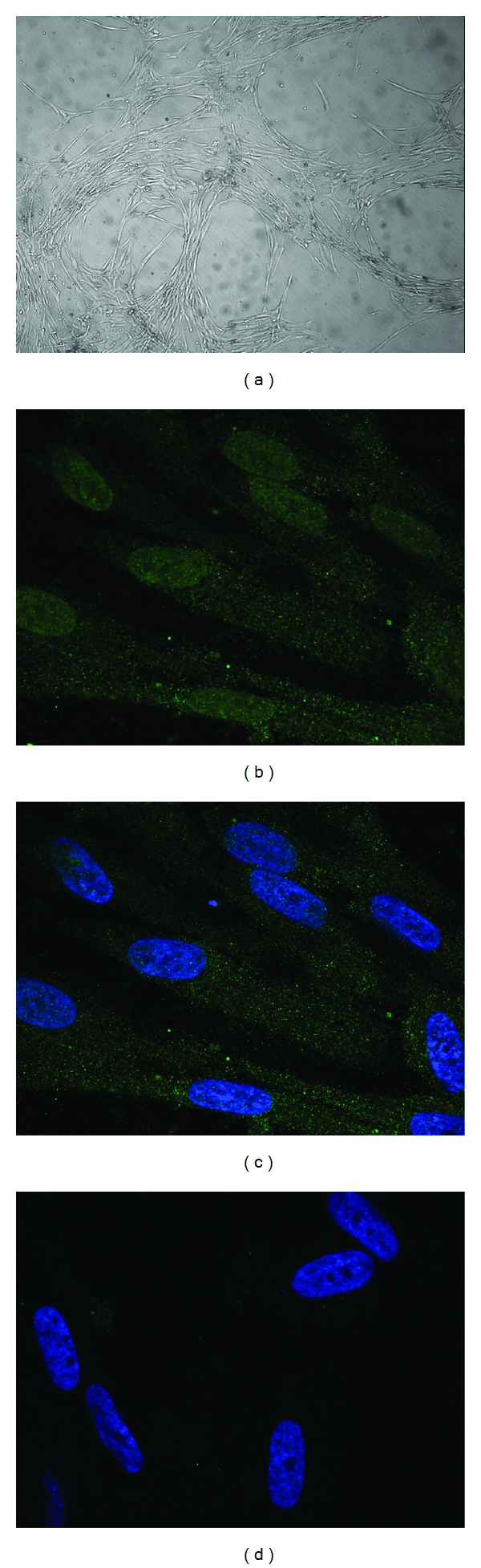
Myogenic differentiation. Phase contrast microscopy of cultures grown in myogenic medium for 21 days (a) at low magnification, immunofluorescence for myogenin in cells cultured in myogenic medium for 21 days; (b) myogenin stained with primary antibody against and with a secondary antibody anti-mouse (conjugated with Alexa Fluor 488); (c) merged with DAPI (blue, nuclei); (d) negative control with DAPI and secondary antibody only.

Myogenic commitment was observed in cell cultures (Figure 6) after 21 days. At immunofluorescence, cells from all three UCs were positive for antimyogenin antibody. Positivity was observed predominantly in the nuclei and lesser in the cytoplasm, according to the literature [20].

**Figure 7:**
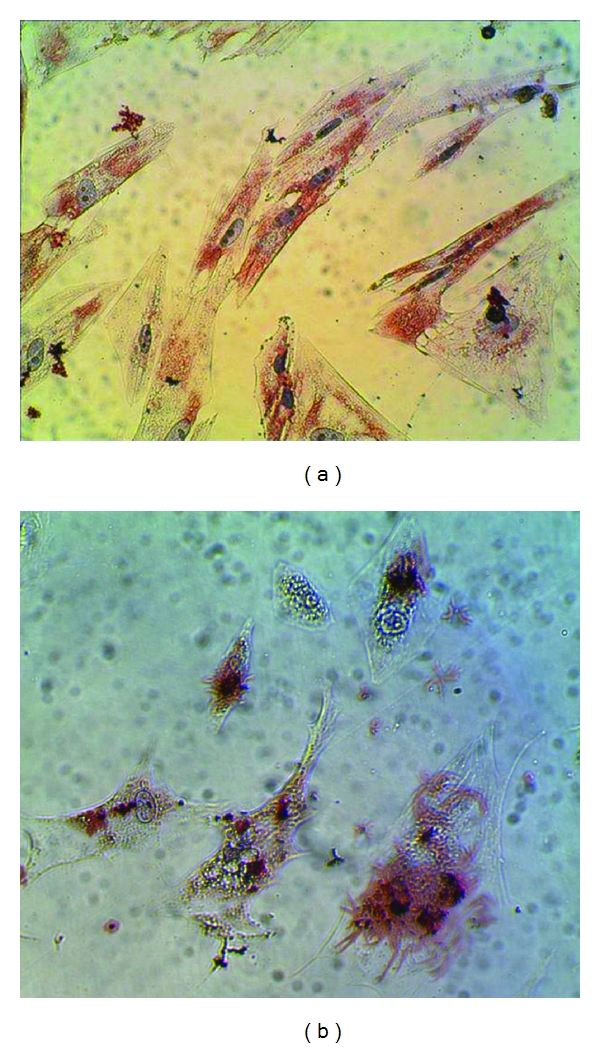
Adipogenic differentiation. Oil Red O staining of adipogenic differentiation of UC-MSCs (a) with formation of smaller lipid vacuoles and of BM-MSCs (b) with formation of large vacuoles.

In the adipogenic-stimulated cultures (Figure 7), UC-MSCs showed lipid deposition and changes in the cellular morphology after 21 days. In all cultures from three UCs, intracellular lipid granules staining positive with Oil Red O were detected after 3 weeks. At variance with bone marrow derived adipocytes, that show larger vacuoles, in our culture UC-MSCs showed smaller lipid vacuoles, possibly related to brown fat commitment.

### 3.3. Fluorescence In Situ Hybridization of UC-MSCs

Cytogenetic analysis of UC-MSCs was performed in two cases of male newborns. The method showed that UC-MSCs were mainly XY (95% and 100%, resp.). This is consistent with a prevalent newborn origin of these cell populations.

### 3.4. Telomere Length Analysis

**Figure 8:**
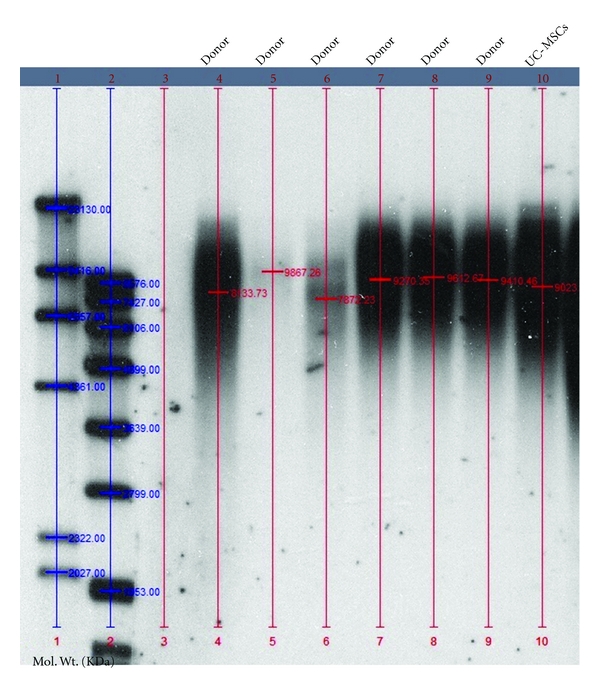
Telomere length of UC-MSCs compared to telomere length of BM-MSCs from 6 different donors (age 20–30) as assayed by Southern Blot analysis of DNA restriction fragments.

Telomere length of UC-MSCs from one UC was determined and compared to telomeres of BM-MSCs at P1 taken from 6 adult volunteers (age 20–30) (Figure 8).

No significant difference was observed between the two cell population. Median value of UC-MSC telomere was 9023 base pairs (9,023 kbp), while median value from all 6 donors was 9340 base pairs (range 7,872–9,867 kbp).

## 4. Discussion

In this study, we apply an easy, reliable, and repeatable method to isolate a mixed population of UC-MSCs from umbilical cord fragments. This protocol was based on simply mincing UCs directly in the MSC expansion medium with minimal mechanical manipulation. We did not remove any blood vessels, and we did not use any enzymatic digestion or any additional purification steps in order to avoid the possible selection of cellular subpopulations. With this feasible method, we collected an adequate number of UC-MSCs already at P1. At immunophenotypic characterization, cells at P1 were positive for the major MSC markers (CD73, CD90, CD105, CD44, and CD29) and negative for the typical haematopoietic marker CD34. Furthermore, we did not find HLA class II in all cells, and we have also observed the presence of a peculiar subpopulation of double negative (HLA-I and HLA-II) UC-MSCs. UC-MSCs obtained with this protocol seem to have a newborn origin and are capable to be committed towards multiple lineage as bone, fat, cartilage, and muscle. Telomere length was similar to that of BM-MSCs taken from young donors. Taken together, all these observations suggest that collecting UC-MSCs at P1 from minced umbilical cord fragments allows to achieve a valuable population of UC-cells that could be used for orthopaedic tissue engineering applications.

In orthopaedics, cell therapy is widely used to enhance tissue repair in different pathologic conditions involving long bone defects or osteochondral lesions. Different cell sources are proposed by tissue engineering, as autologous bone marrow aspirate or allogeneic cells (i.e., allogeneic BM-MSCs or allogeneic chondrocytes). These cells are usually loaded onto suitable scaffolds and directly transferred to the lesion site. This “one-stage” approach eliminates patient's own cell “in vitro” expansion and is considered less expensive than traditional autologous cell culture and implantation, especially in the field of cartilage repair [21].

Autologous bone marrow cells and bone marrow concentrate are regarded as the “gold standard” for bone and cartilage repair [22, 23]. Nevertheless, donor site morbidity is a drawback of this cell source. Additionally, proliferative and differentiation capacity of BM-MSCs are shown to decline with increasing patient's age [24]. Moreover, in bone marrow concentrate, a small number of MSCs are available, thus reducing the efficacy of the cell delivery.

Allogeneic bone marrow could be considered as a solution for these limitations [25], but reduced availability of this resource and decline in BM donations [26] make this solution impractical for large-scale clinical use in orthopaedics.

UC has been recently introduced as a potential alternative to BM in musculoskeletal tissue engineering. Many hypothetical advantages make UC an interesting source of cells. UC is readily available in great quantity, as it is usually discarded during both normal vaginal delivery or cesarean sections. Collecting this source of cells implies no invasive procedure and low costs. Being UC an extraembryonic tissue usually abandoned at the end of the delivery, few ethical problems and legal concerns are involved in this procedure, provided that a complete and informed written consent is obtained from the mothers. Recent studies have shown that MSCs can be obtained from all different compartments of UC, as the Wharton's jelly [10], the perivascular regions [27], and the subendothelium and endothelium areas of UC vessel [11]. All these works have also shown the multilineage potential of these cell populations. Greater expansion capability and long telomere sequences have been observed in UC-MSCs, suggesting a late onset of senescence of this cell population compared to BM-MSCs during in vitro expansion [28]. Immunosuppressive capacity of UC-MSCs and the absence of tumorigenic potential and cytogenetic abnormalities of this cells, when expanded in culture or implanted in vivo, have been extensively described in previous studies [26, 29]. Finally, the theoretical possibility to combine UC-MSCs from multiple donors increases the availability of this source of cells and might be beneficial when a great number of cells are required for a single procedure [30, 31]. All these observations confirm the attractive therapeutic potential of UC-MSCs.

In this study, we have applied an easy and rapid method to collect an adequate number of UC-MSCs, by simply mincing the UCs and cultivating the small UC fragments and the migrating MSCs for a total of 6 weeks in a standard culture medium enriched with human platelet lysate and fetal bovine serum.

Previously reported methods have described the isolation of MSCs from UC through multiple steps in order to select a specific cell source. In 2003, Mitchell et al. introduced a technique to collect multipotent stem cells from Wharton jelly by removing all vascular network [32]. In 2004, Wang et al. proposed to scrape off the Wharton jelly from the whole UC and to perform an enzymatic digestion with collagenase and trypsin [33]. Recently, Montanucci et al. [34] demonstrated a procedure involving multiple enzymatic digestion with hyaluronidase and human recombinant Liberase of UC samples and centrifugation of the digestion product to cleave the cells out of the original Wharton jelly matrix. Complex methods are also required to isolate human umbilical cord perivascular cells, implying tissue dissection in order to isolate UC vessel, collagenase digestion, centrifugation, and magnetic bead depletion protocol [27]. In all these studies, selected cell populations were obtained with detailed procedures that, albeit effective, implied non-negligible tissue manipulation. All these procedures may be considered too complicated for a clinically oriented large-scale cell therapy.

These observations encouraged us to apply a simple, practical, and economic method to rapidly obtain a mixed MSC population from minced umbilical cord, similarly to recent studies [26, 30].

In order to reduce the risk of external contamination, only UCs from cesarean sections were utilized. In our opinion, UCs from cesarean sections are more suitable for tissue engineering than UCs from vaginal delivery, due to the possibility to collect these samples in the clean environment of the operating room.

The method described in this study has some practical advantages. It allows to cut off time-consuming steps involving the use of enzymatic solution and the need for long incubating period. It involves a minimal tissue manipulation consisting in mechanically mincing the tissue. This principle does not impair the vitality of the tissue, as shown in previous work involving cartilage biopsies [35] and UC samples [30]. Moreover, a mixed MSC population is obtained with our method, in order to preserve the “mesenchymal properties” of all UC compartments.

With our protocol, an adequate number of cells was obtained to perform all studies from each UC without multiple expansion passages. This study was not designed to primarily obtain large number of cells, harvesting the maximum number of UC-MSCs from each cord as a clinical use should require. This method was aimed to extract a consistent number of cells with minimal manipulation for a preliminary in vitro pilot study. Indeed, the natural tendency of MSCs to attach to plastic dishes was the main element of our separation technique. For this reason, a great amount of umbilical cord was discarded after 15 days of incubation and not used to obtain more MSCs. The final number of cells, albeit not exceedingly high, was nevertheless sufficient for the whole design of the experiment including the cell characterization and the commitment toward osteoblastic or chondroblastic or myoblastic or adipoblastic line. In light of an in vivo application, these methods could be anyway suitable, because the umbilical cord is a virtually unlimited source of cells normally discarded after birth and the extraction efficiency can be therefore a secondary problem when the primary source of cells is widely available at no costs. We are nevertheless aware that processing the whole umbilical cord with different harvesting procedures and different method of mincing and expanding the cell population would theoretically lead to a greater number of cells available from each UC.

The MSCs collected showed a fibroblast-like morphology, when adherent to the plastic dishes. At the immunophenotypic characterization, cells exhibited a phenotype similar to that of BM-MSCs, being positive for the main MSC markers (CD73, CD90, CD105), for CD44, CD29, and HLA class I and negative for the haematopoietic marker CD34 and for HLA class II, in agreement with other reports [36]. Interestingly, a peculiar population of UC-MSCs negative both for HLA class I and HLA class II was found. This “double negative” cell population seems particularly suitable for an allogeneic use. Further studies will aim to properly separate these specific cells in order to apply them in future in vivo experiments. The mixed UC-MSC population obtained with this method was shown to have a newborn origin. This can partially explain the great plasticity of these cells.

Indeed, we obtained osteogenic, adipogenic, chondrogenic, and myogenic early commitment after culture with differentiation media. We are aware that we did not reached a morphology similar to differentiated tissues, but rather we observed a commitment toward a specific cell type or, in other words, a progression toward an osteoblastic or chondroblastic or myoblastic or adipoblastic line. Moreover, we still observed differences in the plasticity of these cells compared to BM-MSCs, that show a more advanced differentiation stage in similar culture conditions (see comparative Figures 4, 5, 6, and 7).

Published data on UC-MSC differentiation potential are still controversial; our results are in contrast with some previously reported observations showing low capacity of UC-MSC to differentiate towards bone, adipocytes, and chondrocytes [15, 26, 37]. We believe that the osteogenic, adipogenic, and chondrogenic commitment obtained in this study may be related to the specific composition of the medium used in this protocol and possibly could be further improved. These early commitment stages could be further enhanced by the influence of the in vivo microenvironment to complete the differentiation process. 

For all these reasons, these cells could be considered a putative candidate for cell therapy in orthopaedic tissue engineering.

We have also assessed the length of the telomere in UC-MSCs collected with this method, as indicator of cell replication history and senescence, and we have compared the result with the telomere length of BM-MSCs obtained from healthy young donors. We observed analogous results in the two different cell populations. This is in agreement with the literature [28] and shows that the mixed UC-MSCs population at P1 obtained with this method share an equivalent proliferative potential with MSCs from BM aspirate of young donors. Nonetheless, it also suggests that the procedure of cell isolation described in our study does not induce substantial cell senescence in the UC-MSCs.

## 5. Conclusion

In conclusion, UC-MSCs can be obtained after a primary culture at P1 with this simple and rapid method. This mixed cell population of predominant newborn origin has shown signs of osteogenic, adipogenic, and chondrogenic commitment along with long telomere sequences suggestive for a high proliferative potential. Thus, UC-MSCs at P1 seem to have the potential to be good candidates for tissue engineering applications in orthopaedics. The concept of this study may indeed be considered as a future hypothetical option for patients who might benefit from stem cells therapy. However, given these preliminary results, testing in vivo the regenerative potential of this cell population in an animal models, including large animals, will be the next logical step.
